# Sea Buckthorn Oil as a Valuable Source of Bioaccessible Xanthophylls

**DOI:** 10.3390/nu12010076

**Published:** 2019-12-27

**Authors:** Cristina Tudor, Torsten Bohn, Mohammed Iddir, Francisc Vasile Dulf, Monica Focşan, Dumitriţa Olivia Rugină, Adela Pintea

**Affiliations:** 1 Faculty of Veterinary Medicine, University of Agricultural Sciences and Veterinary Medicine, Mănăştur Street, 3-5, 400372 Cluj-Napoca, Romania; cristina.tudor@usamvcluj.ro (C.T.); francisc_dulf@yahoo.com (F.V.D.); dumitrita.rugina@usamvcluj.ro (D.O.R.); 2 Department of Population Health, Luxembourg Institute of Health, Nutrition & Health Research Group, 1A-B, rue Thomas Edison, L-1445 Strassen, Luxembourg; torsten.bohn@lih.lu (T.B.); mohammed.iddir@lih.lu (M.I.); 3 Nanobiophotonics and Laser Microspectroscopy Center, Interdisciplinary Research Institute on Bio-Nano-Sciences, Babes-Bolyai University, Treboniu Laurean Street, 42, 400271 Cluj-Napoca, Romania; iosinmonica@yahoo.com

**Keywords:** bioaccessibility, carotenoids, simulated digestion, food matrix, apolar secondary plant compounds, sea buckthorn oil

## Abstract

Sea buckthorn oil, derived from the fruits of the shrub, also termed seaberry or sandthorn, is without doubt a strikingly rich source of carotenoids, in particular zeaxanthin and β-carotene. In the present study, sea buckthorn oil and an oil-in-water emulsion were subjected to a simulated gastro-intestinal in vitro digestion, with the main focus on xanthophyll bioaccessibility. Zeaxanthin mono- and di-esters were the predominant carotenoids in sea buckthorn oil, with zeaxanthin dipalmitate as the major compound (38.0%). A typical fatty acid profile was found, with palmitic (49.4%), palmitoleic (28.0%), and oleic (11.7%) acids as the dominant fatty acids. Taking into account the high amount of carotenoid esters present in sea buckthorn oil, the use of cholesterol esterase was included in the in vitro digestion protocol. Total carotenoid bioaccessibility was higher for the oil-in-water emulsion (22.5%) compared to sea buckthorn oil (18.0%) and even higher upon the addition of cholesterol esterase (28.0% and 21.2%, respectively). In the case of sea buckthorn oil, of all the free carotenoids, zeaxanthin had the highest bioaccessibility (61.5%), followed by lutein (48.9%), making sea buckthorn oil a potential attractive source of bioaccessible xanthophylls.

## 1. Introduction

Over the last decades, the carotenoid content and composition in various fruits and vegetables have been extensively investigated. Most studies have been focused on provitamin A carotenoids (particularly β-carotene), which, following human digestion and absorption, can be enzymatically cleaved to yield vitamin A [1,2,3]. Besides the provitamin A carotenoids, other members of the carotenoid family are relevant for the human body by exerting specific functions. For instance, lutein and zeaxanthin accumulate in the macular region of the human retina and, in addition to the proven importance of vitamin A in the normal functioning of the visual system, the two dihydroxy-xantophylls are considered to offer protection against free-radical and blue light induced damage that can cause several eye diseases, such as age-related macular degeneration and cataracts [4,5]. Furthermore, it has been proposed that they may improve cognitive function [6]. In the human diet, lutein is the dominant xanthophyll, and even though some good few sources of zeaxanthin are known such as egg yolk, corn and orange pepper [7,8], the usual dietary ratio of lutein to zeaxanthin is approximately 5:1 [9]. Sea buckthorn oil is of special interest for human nutrition, because of the fruits’ high content of carotenoids, in particular zeaxanthin (present in an esterified form with one or two fatty acids), and can be considered a noteworthy contributor of this ophthalmo-protective xanthophyll to dietary intake [10].

Despite being widespread and abundant in many food products, knowledge about the human digestion and absorption of carotenoids is limited and a growing interest in the bioaccessibility (i.e., the fraction of ingested compounds that becomes available for uptake by absorptive cells of the small intestine) of these naturally occurring bioactive compounds has been observed lately. In brief, their absorption requires the solubilization of the released carotenoids from the food matrix into a lipid phase after ingestion or during processing and the successful enzymatic digestion of this phase. A major obstacle in the bioaccessibility of carotenoids is represented by their entrapment in complex structures in different carotenoid-containing foods. Their impeded release subsequently leads to a poor incorporation within the mixed micellar fraction during the intestinal digestion, which further translates into a very low absorption of these bioactive compounds. Food processing techniques such as mincing, pureeing, steaming, and the addition of fat facilitate the transfer of carotenoids from the food matrix to lipid droplets during the gastric phase and have been proven to substantially enhance the bioaccessibility of carotenoids [11]. Moreover, oil-in-water emulsions are more readily digested than individual oil droplets, and carotenoid bioaccessibility is considered to be higher from these systems because of the small particle size and larger active surface area [12]. Carotenoid bioaccessibility can also be considerably reduced by the presence of high amounts of fiber in the food matrix [13,14,15] and by the presence of high concentrations of divalent minerals [16].

In the last two decades, several simulated gastro-intestinal digestion models have been proposed in the literature for the assessment of carotenoid bioaccessibility, which differ significantly in several aspects, such as the number of digestion steps included in the digestion model, type, source and concentration of the enzymes used, pH, digestion time, salt concentrations etc. This has made it impossible to compare the results from different research groups, as each in vitro model could have its own digestive conditions applied. For this reason, Minekus et al. [17] proposed a standardized static in vitro digestion model that has since been successfully used in numerous studies, also involving carotenoid-containing foods [18,19,20].

To the best of our knowledge, the bioaccessibility of carotenoids from sea buckthorn oil has not yet been investigated. For this purpose, in the current study, a physiologically standardized in vitro digestion protocol [17,21] was applied in order to investigate the bioaccessibility of carotenoids from cold-pressed sea buckthorn oil as compared to the bioaccessibility of carotenoids from an oil-in-water emulsion prepared with the use of the above-mentioned oil.

## 2. Materials and Methods

### 2.1. Reagents

The cold-pressed oil, a kind gift from a local producer from Bihor County (in the Northern region of Romania), was obtained by processing sea buckthorn berries, more precisely Mara variety. The oil was stored at room temperature, away from humidity and light, until use (typically within 15 days).

Tween-20 (Art. No. P1379), pepsin from porcine gastric mucosa (Art. No. P6887), pancreatin from porcine pancreas (Art. No. P7545), cholesterol esterase from porcine pancreas (Art. No. 26745) and bovine bile extract (Art. No. B8631) were purchased from Sigma-Aldrich (Steinheim, Germany).

β-Carotene, β-cryptoxanthin, and zeaxanthin standards were purchased from Extrasynthese (Lyon, France), while zeaxanthin monopalmitate, zeaxanthin dipalmitate, and β-cryptoxanthin palmitate were obtained by semi-synthesis and purified by HPLC-PDA, as previously reported [22,23].

All used chemicals and reagents were of analytical or HPLC grade. The water used for all experiments was treated in a Milli-Q water purification system.

### 2.2. Emulsion Formation and Characterization

A sea buckthorn oil-in-water (o/w) emulsion was prepared with a 0.1 surfactant-to-oil ratio, according to the method described by Kiokias and Oreopoulou [24]. Briefly, 9 mL water and 0.1 g Tween-20 (used as emulsifier) were place in a tube. The cold-pressed sea buckthorn oil (1 g) was added dropwise and the sample (kept on ice-cold water) was sonicated (Vibra Cell, Sonics & Materials Inc., Newtown, CT, USA) for 6 min (amplitude 75%). The stable emulsion was kept at 4 °C, protected from light and oxygen, and used within five days for the experiments. The emulsion was characterized in terms of particle size distribution and ζ-potential employing a Zetasizer NanoZS90 system (Malvern Instruments Ltd., Malvern, UK) equipped with a He-Ne laser operating at 633 nm (5 mW), and further subjected to a simulated gastrointestinal digestion. Specifically, the hydrodynamic diameter and ζ-potential analysis were performed at a scattering angle of 90° and temperature of 25 °C. The emulsion was measured three times and the mean values are reported.

### 2.3. In Vitro Digestion

The static in vitro digestion model described by Minekus et al. [17] was amended and applied for the carotenoid bioaccessibility determination from sea buckthorn oil and oil-in-water emulsion. Taking into account the short residence time in the oral cavity, both the sea buckthorn oil and oil-in-water emulsion were subjected to a two-phase in vitro digestion consisting of gastric and small intestinal phases. During normal in vivo human digestion, a large amount of zeaxanthin esters are converted into free zeaxanthin [25,26], and several protocols were adapted so as to maximize the cleavage of carotenoid esters with the use of different enzymes such as cholesterol esterase (CEL). Although the addition of CEL in the intestinal phase was not included in the in vitro digestion model proposed by Minekus et al. [17], it was included in the present in vitro digestion model as 1 U of CEL per mL in the final digestion mixture, as suggested by Wen et al. [27].

#### 2.3.1. Gastric Phase

An aliquot of 0.1 g sea buckthorn oil (1 g oil-in-water emulsion) in 6.15 mL water was combined with 4 mL of simulated gastric fluid (SGF), 1 mL of porcine pepsin solution in SGF (2000 U/mL in final digestion mixture) and 31 μL of CaCl_2_ (0.03 M). HCl (1 M) was added to reduce the pH to 3.0 and water was added to a final volume of 12.5 mL. The mixture was homogenized and incubated at 37 °C for 2 h (95 rpm) in a shaking incubator (New Brunswick Innova 44, Eppendorf AG, Hamburg, Germany).

#### 2.3.2. Intestinal Phase

The gastric chyme was mixed with 6 mL simulated intestinal fluid (SIF), 2 mL porcine pancreatin solution in SIF (1000 U/mL in final digesta, in lipase activity), 2 mL bile extract solution in SIF (10 mM in final digestion mixture) and 250 µL CaCl_2_ (0.03 M). Porcine CEL was added as 1 U/mL in final digesta to investigate its ability to hydrolyze carotenoid esters in this in vitro digestion model. The pH was adjusted to 7 using 1 M NaOH and water was added to a final volume of 25 mL. The mixture was homogenized and incubated at 37 °C for 2 h in a shaking incubator (95 rpm).

At the end of the intestinal phase, the digesta was immediately centrifuged for 60 min at 4800 g and 4 °C (Eppendorf 5810 R, Eppendorf AG, Hamburg, Germany) to minimize the enzyme activity and to remove the undigested material. The supernatant considered to contain the carotenoids released from the food matrix (i.e., the liberated carotenoids) was afterwards membrane-filtered (0.2 μm nylon filter) in order to separate the micellar fraction (i.e., the bioaccessible fraction).

### 2.4. Carotenoid Extraction and HPLC-DAD Analysis

An aliquot of 2 mL of the mixed micellar fraction was combined with 4 mL of hexane:acetone (1:2, *v*/*v*), vortexed, and centrifuged for 2 min at 3200× *g* at 4 °C. The upper organic phase was collected and the lower phase was re-extracted 2 times with 4 mL hexane. The organic extracts were combined, evaporated to dryness with the use of a rotary evaporator (Heidolph, Heidolph Instruments GmbH & CO, Schwabach, Germany) and stored at −20 °C until HPLC analyses.

For HPLC analyses, the samples were dissolved in diethyl ether in amber vials. HPLC separation of carotenoids was carried out using a Shimadzu LC20 AT high performance liquid chromatograph (Shimadzu Corporation, Kyoto, Japan) with a SPDM20A diode array detector and an YMC C30 reversed phase column (250 × 4.6 mm i.d., 3 μm particle size). The mobile phase consisted of methanol/tert-butyl methyl ether/water (83:15:2, *v*/*v*/*v*) as eluent A and tert-butyl methyl ether/methanol/water (80:7:2, *v*/*v*/*v*) as eluent B, using a gradient program as follows: 0 min 0% solvent B, 20 min 0% B; 130 min 82% B; 132 min 0% B, followed by equilibration of column for 10 min. The flow rate was fixed at 0.8 mL/min. The DAD operated in the range of 300–600 nm for the acquisition of UV-Vis spectra, while the chromatograms for quantitative analysis were extracted at 450 nm.

Individual carotenoids were identified by comparing their retention time, UV-Vis spectra (λ_max_, spectral fine structure, *cis* peak intensity) with those of the available standards, and with our previous results and literature data. Quantitative analysis was performed using external calibration with β-carotene, β-cryptoxanthin, and zeaxanthin (1–200 μg/mL).

### 2.5. GC-MS Analysis of Fatty Acids

Fatty acid methyl esters (FAMEs) were obtained from total lipids using the acid-catalyzed transesterification procedure described by Christie [28]. The FAMEs were analyzed by gas chromatography-mass spectrometry (GC-MS) using a PerkinElmer Clarus 600 T GC-MS (PerkinElmer, Inc., Shelton, CT, USA) fitted with a Supelcowax 10 (60 m × 0.25 mm i.d., 0.25 μm film thickness; Supelco Inc., Bellefonte, PA, USA) capillary column, using helium as a carrier gas (flow rate of 0.8 mL/min) [29]. The temperature program for the column was: initial temperature, 140 °C, increase by 7 °C/min to 220 °C, and hold for 23 min. The injection volume was 0.5 μL (split ratio of 1:24) and the injector was set at 210 °C. The MS operating conditions were as follows: electron impact ionization voltage 70 eV (E.I., positive ion electron impact mode), trap current of 100 μA, source temperature of 150 °C, scan rate 0.14 scan/s and scanned mass range 22–395 m/z. The identification of FAMEs was achieved by comparing their retention times with those of known standards (37 component FAME Mix, SUPELCO # 47885-U) and the resulting mass spectra to those in our database (NIST MS Search 2.0). The compositions of fatty acids in the studied lipids were expressed as percentages (%) of the total FAME peak areas. All experiments were performed in triplicate.

### 2.6. Calculation and Statistical Analysis

In vitro bioaccessibility (percentage of bioaccessible carotenoids) was calculated as the amount of the bioactive compound transferred into the micellar phase, in relation to the total content found in the non-digested food sample, i.e., cold-pressed sea buckthorn oil. Zeaxanthin dipalmitate (ZDP) hydrolysis efficiency was determined as the molar ratio of free zeaxanthin in the final digestion mixture to zeaxanthin dipalmitate before the in vitro digestion [27].

Statistical analysis was done using unpaired t test with Welch’s correction of Graph Pad Prism version 6.00. Analyses were performed in triplicate and values are given as mean ± SD (* significant *p* < 0.05, ** very significant *p* < 0.01, ^###^ extremely significant *p* < 0.001) versus control (sea buckthorn oil before in vitro digestion).

## 3. Results and Discussion

The cold-pressed sea buckthorn oil was characterized in terms of carotenoid and fatty acid composition and further used for the preparation of the oil-in-water emulsion. Most of the compound profiles were in agreement with literature data.

### 3.1. Carotenoid Composition

The total content of carotenoids in sea buckthorn berries was found to be relatively high (Table 1) in comparison to other fruits [25]. Studies have shown significant differences in terms of the composition and content of carotenoids, which can be explained by the genetic variation, geographic location, growing conditions, stage of maturity when harvested, storage conditions, and methods of analysis [30,31].

Being cold-pressed, the sea buckthorn oil used in the present study had a high content of total carotenoids. Fourteen carotenoids were identified and lutein, zeaxanthin, β-carotene and α-carotene represented the free carotenoid fraction (peaks 1–5). Free and esterified forms (with one or two fatty acids) of zeaxanthin corresponded to 76.35% of the identified carotenoids (peaks 2, 6, 7, 11–14). As shown in Table 1, the most abundant compound was zeaxanthin, mainly in its esterified form with two molecules of palmitic acid, zeaxanthin dipalmitate (peak 14). During ripening, xanthophylls such as zeaxanthin develop into esterified forms which constitute more stable forms of carotenoid storage in fruits [32].

Pop et al. [33] investigated the carotenoid composition of berries from six Romanian sea buckthorn varieties, and the total carotenoid content ranged between 53 and 97 mg/100 g dry weight, with zeaxanthin dipalmitate (ZDP) present in the highest amount. In another study, three sea buckthorn species from the cold deserts of the Himalayas were characterized and the total carotenoid content ranged from 692 to 3420 mg/kg [34]. Depending on the origin of the berries, the total carotenoid content in oil can reach 314–2139 mg carotenoids/100 g in sea buckthorn oil from China, and 900–1000 mg/100 g in sea buckthorn oil from the Pamir region [35].

### 3.2. Fatty Acid Composition

**Figure 1:**
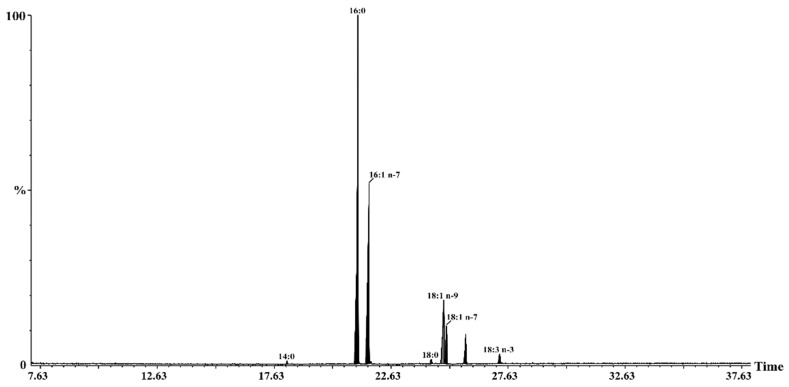
GC-MS chromatogram of FAMEs in sea buckthorn oil analysed on a Supelcowax 10 capillary column. Peak identification: (14:0), myristic; (16:0), palmitic; (16:1 n-7), palmitoleic; (18:0), stearic; (18:1 n-9), oleic; (18:1 n-7), *cis*-vaccenic; (18:2 n-6), linoleic; (18:3 n-3), α-linolenic acids.

Sea buckthorn oil has a special fatty acid composition compared to other vegetable oils [36]. Eight fatty acids have been identified in the cold-pressed sea buckthorn oil (Table 2 and Figure 1). The most abundant saturated fatty acid was palmitic acid, while palmitoleic and oleic acids were the dominating monounsaturated fatty acids. Palmitoleic acid (16:1 n-7) is a taxonomic marker of sea buckthorn oil and its presence in high amounts is rare among plant oils [34]. Bialek et al. [36] investigated the fatty acid profile of unconventional plant oils for cosmetic use and found a high amount of palmitoleic acid (29.4%) in sea buckthorn oil. In addition, the content of SFA, MUFA, and PUFA found were 33.5%, 51.0% and 5.2%, respectively. The percentage of palmitoleic acid in sea buckthorn berries (pulp) ranged between 13.35 and 36.68% in various Romanian cultivars [37], while in selected locations from India and Sweden it ranged between 31.9 and 43.3% [31]. Sea buckthorn seed oil has been shown to have a higher amount of PUFA (65–72%) than pulp oil (3–7%) [38].

By using oil and a carotenoid-rich oil-in-water emulsion as food matrices, the major limiting factor represented by the release of carotenoids from the food matrix was removed. Another key factor in carotenoid bioaccessibility is represented by the presence of dietary fat in the small intestine which stimulates the release of bile acids [39]. Moreover, lipids act as an ideal carrier of these phytochemicals, and carotenoid bioaccessibility was previously found to be enhanced by their presence [40].

Several studies have shown that dietary sources rich in MUFA could lead to an increased carotenoid bioaccessibility [41] and absorption [42,43] compared to sources rich in SFA and PUFA. In our case, MUFA represented 44.24% of total fatty acids, making sea buckthorn oil a promising source of bioaccessible carotenoids.

### 3.3. Sea Buckthorn Oil-in-Water Emulsion

**Figure 2:**
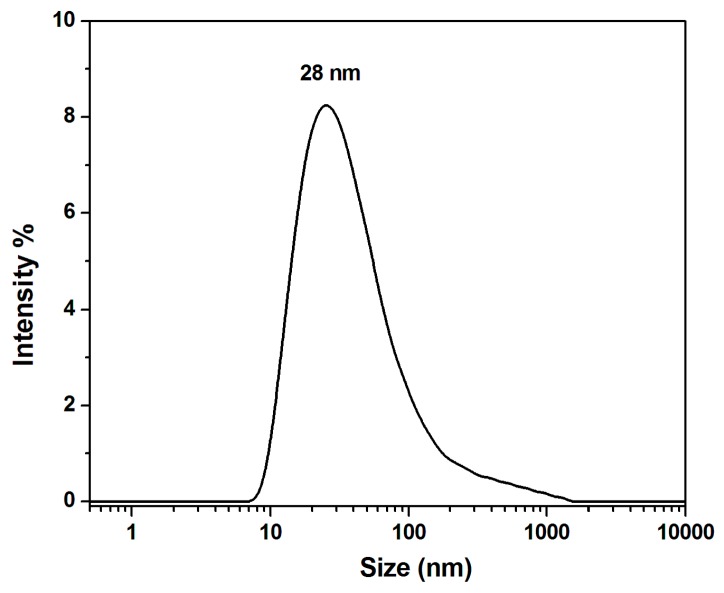
Dynamic light scattering (DLS) analysis of the sea buckthorn oil-in-water emulsion.

Systems such as oil-in-water emulsions, in which oil droplets are dispersed in an aqueous phase, can constitute a successful and effective strategy to enhance carotenoid bioaccessibility and absorption. These systems are considered better carriers for lipophilic compounds such as carotenoids. Figure 2 illustrates the dynamic light scattering data of the emulsion, presenting a hydrodynamic diameter of 28 nm with a standard deviation of ±1.7 nm, recorded for *n* = 3 measurements (polydispersity index 0.310). In addition, their surface is negatively charged (−15 mV), as proven by the ζ-potential analysis.

### 3.4. Bioaccessibility of Carotenoids from Sea Buckthorn Oil and Sea Buckthorn Oil-in-Water Emulsion

By using the in vitro digestion protocol proposed by Minekus et al. [17], carotenoid esters are partially hydrolyzed during the intestinal passage by the small amount of CEL present in pancreatin [44]. However, in the case of samples that contain a high amount of carotenoid esters such as sea buckthorn oil, the hydrolysis of xanthophyll esters is a necessary requirement for a more accurate determination of carotenoid bioaccessibility [8]. In this respect, porcine CEL was included as 1 U per mL in the final digestion mixture in the in vitro digestion model of both cold-pressed oil and oil-in-water emulsion, based on its previously proven ability to participate in the hydrolytic process of carotenoid esters [45].

According to the standardized static in vitro method, if the food contains high amounts of lipids, a concentration of 2000 U/mL pancreatic lipase activity should be reached in the final digestion step during the intestinal phase. In our protocol, the concentration used was 1000 U/mL of lipase activity, due to the low solubility of pancreatin at such high concentrations and based on the findings of Wen et al. [27] that 1000 U/mL pancreatin along with 1 U/mL CEL should be sufficient for ZDP hydrolysis.

**Figure 3:**
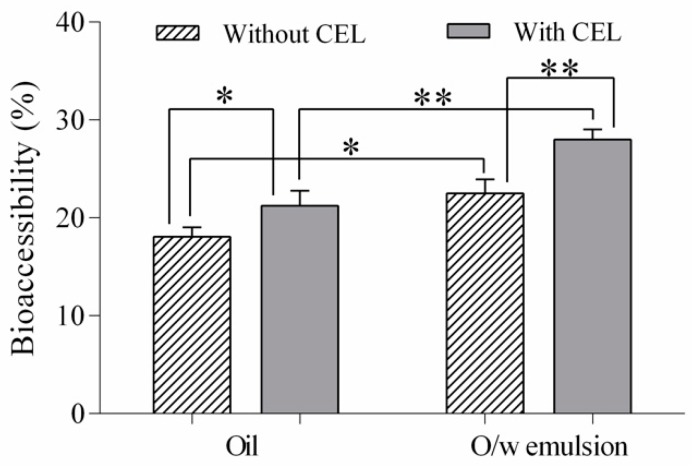
Total carotenoid bioaccessibility of sea buckthorn oil and oil-in-water emulsion (o/w) with and without the addition of cholesterol esterase in the in vitro digestion protocol. Values are given as mean ± SD (* significant *p* < 0.05, ** very significant *p* < 0.01).

As shown in Figure 3, the highest total carotenoid bioaccessibility was observed in the case of the oil-in-water emulsion subjected to in vitro digestion with the addition of cholesterol esterase (27.97%), and the lowest was observed for the oil after the in vitro digestion without the addition of cholesterol esterase (18.04%). These results are rather high compared to other studies reported in the literature on the bioaccessibility of carotenoid-containing plant sources. Kaulmann et al. [46] investigated the bioaccessibility of carotenoids from four varieties of Brassicaceae and Prunus and found the highest total carotenoid bioaccessibility of 11%. Rodrigues et al. [18] compared four protocols of in vitro digestion for the assessment of carotenoid bioaccessibility in murici (*Byrsonima crassifolia*), an Amazonian fruit rich in lipids, and the highest overall bioaccessibility (22%) was obtained when using an adapted INFOGEST method, which is more consistent with our findings.

**Figure 4:**
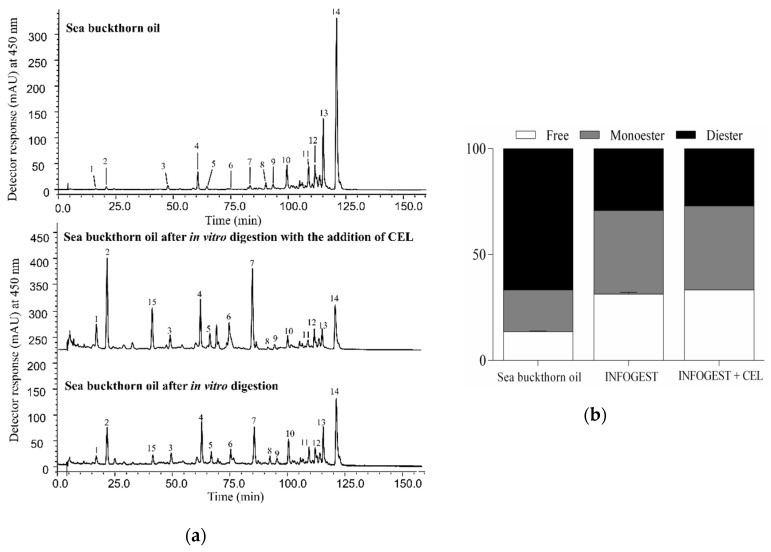
(**a**) Chromatograms of sea buckthorn oil before and after in vitro digestion with and without the addition of CEL. Peak assignments are given in Table 1. (**b**) Percentages of free carotenoids, mono- and di-esters from sea buckthorn oil before and after in vitro digestion with and without the addition of CEL.

The carotenoid profile of sea buckthorn oil (a), as well as the percentages of free carotenoids, mono- and di-esters (b) before and after the in vitro digestion can be seen in Figure 4. The di-ester percentage (composed only of zeaxanthin di-esters, Table 1) decreased from 66.73% in sea buckthorn oil before in vitro digestion to 29.26% after gastrointestinal digestion, and to 27.04% upon supplementation of the protocol with 1 U per mL of cholesterol esterase. Furthermore, free carotenoid percentage increased from 13.60% to 31.28% and 33.26% respectively.

**Figure 5:**
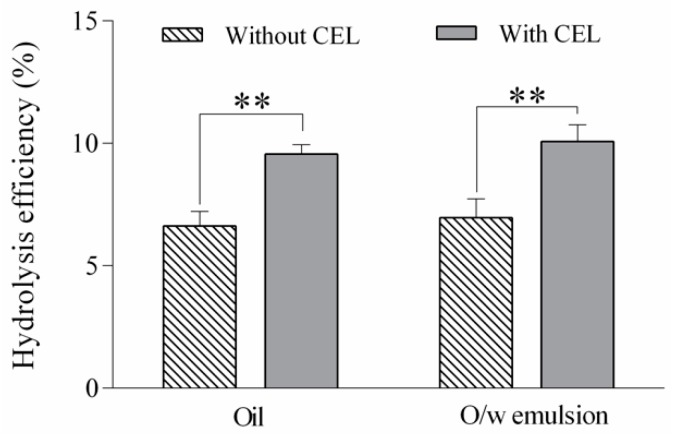
Hydrolysis efficiency of zeaxanthin dipalmitate from sea buckthorn oil and oil-in-water emulsion (o/w) with and without the addition of cholesterol esterase in the in vitro digestion protocol. Values are given as mean ± SD (** very significant *p* < 0.01).

In zeaxanthin-rich fruits such as sea buckthorn or goji berries, zeaxanthin dipalmitate constitutes the major carotenoid [20,33,47]. The insufficient hydrolysis of carotenoid esters during the intestinal phase in the in vitro digestion models is a well-known issue and represents one of the major drawbacks of these models. In the present study, the addition of CEL at the concentration of 1 U per mL in final digestion mixture led to the increase of the ZDP hydrolysis efficiency from 6.62% to 9.55% for sea buckthorn oil and from 6.96% to 10.07% in the case of sea buckthorn oil-in-water emulsion (Figure 5). When using the ZDP standard as a test sample, Wen et al. [27] found an increase in hydrolysis efficiency from 4.1% to 17.4% after the standardized in vitro digestion with pancreatin added at 1000 U lipase activity per mL and supplementation with 1 U of CEL per mL, emphasizing the important contribution of this enzyme in ZDP cleavage.

By using sea buckthorn oil as a test sample, and thereby providing a lipid source during the gastrointestinal digestion, the transfer of carotenoids into mixed micelles was facilitated and the bioaccessibility was enhanced. The effect of variations of the lipophilic phase was previously examined by Hempel et al. [20], who tested the addition of coconut fat (rich in SFA) in the digestion of goji berries and found a significant increase in zeaxanthin micellarization (from 6.7 ± 0.9% to 13.3 ± 0.8%). In another study, the addition of coffee creamer (68% of the total fat comprised of SFA) led to the increase of both the hydrolysis efficiency of ZDP (from 3.4% to 15.9%) and bioaccessibility of total zeaxanthin (from 10.6% to 20.8%) [27].

Although a significant increase in xanthophyll esters hydrolysis could be observed, ester forms were still incorporated into mixed micelles along with the free forms (Figure 4b). Zeaxanthin monopalmitate (ZMP) content increased from 3.53% in sea buckthorn oil to 7.39% after the in vitro digestion protocol and to 9.30% upon supplementation with CEL, and in the case of the o/w emulsion to 7.18% after the in vitro digestion and 10.48% after the involvement of CEL. By this, the ability of pancreatic lipase to hydrolyze zeaxanthin di-esters through zeaxanthin mono-esters was emphasized, even without the addition of CEL.

**Figure 6:**
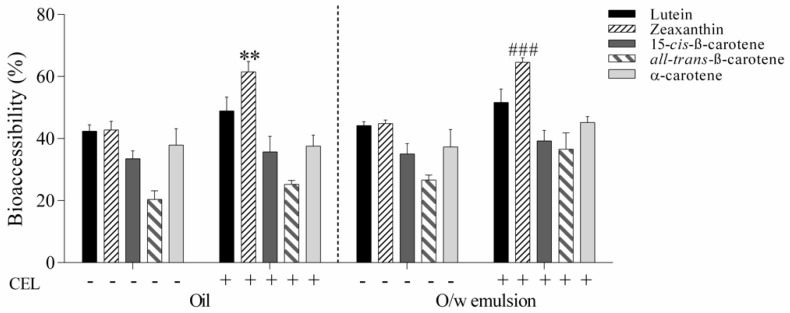
Bioaccessibility of free carotenoids from sea buckthorn oil and oil-in-water emulsion with and without the addition of cholesterol esterase in the in vitro digestion protocol. Carotenoid bioaccessibility was calculated as the percentage of the respective carotenoid transferred from the test food to the micellar phase. Values are given as mean ± SD (** very significant *p* < 0.01, ^###^ extremely significant *p* < 0.001).

In general, the bioaccessibility of xanthophylls has been shown to be higher than that of carotenes [46,48]. In the present study, the high values of xanthophyll bioaccessibility (Figure 6) can be also attributed to the hydrolysis of esters during the simulated digestion model, which yielded more free carotenoids. By using CEL, and thus promoting the hydrolytic reaction, even more free xanthophylls were produced which were incorporated into the micelles and consequently boosted the bioaccessibility.

Regarding free β-cryptoxanthin (peak 15), due to the fact that it was not present in the sea buckthorn oil prior to in vitro digestion, the bioaccessibility could not be determined. However, the presence of free β-cryptoxanthin after the simulated gastrointestinal digestion clearly indicated that not only zeaxanthin esters were subjected to hydrolysis, but also β-cryptoxanthin esters.

In the case of sea buckthorn oil, the bioaccessibility of *all-trans*-β-carotene was 20.36% and for the o/w emulsion 26.65%. Using the COST INFOGEST protocol to investigate spinach carotenoid liberation and in vitro accessibility, and after the addition of fat (butter), [19] found the highest in vitro accessibility of β-carotene 6.2 ± 0.6%. 

Sea buckthorn oil has a unique chemical composition, combining high concentrations of carotenoids with a very particular fatty acid profile and a high content of tocopherols. The most important carotenoid in the Romanian sea buckthorn oil is zeaxanthin, mostly in esterified forms. In the present study we showed that both sea buckthorn oil and oil-in-water emulsion are highly bioaccessible sources of zeaxanthin, β-cryptoxanthin and β-carotene. In this respect, approximately 1.4 g of sea buckthorn oil would provide the AREDS 2 recommended daily intake of zeaxanthin (2 mg) for the prevention of eye diseases such as age-related macular degeneration. Sea buckthorn oil can have a positive impact on cognitive performance in both the elderly and young subjects due to its high content of zeaxanthin [6,49]. Additionally, sea buckthorn oil could improve vitamin A status, providing more bioaccessible β-carotene and β-cryptoxanthin compared to other sources. Palmitoleic acid (16:1 n-7) is an active molecule found in high concentrations in sea buckthorn, macadamia nuts and fish. Although it can be synthesized by humans and it accumulates in the adipose tissue, palmitoleate is considered as a plausible nonpharmaceutical approach to prevent metabolic and inflammatory diseases [50]. Palmitoleic acid has been considered a lipokine since it was found to increase insulin secretion and to suppress hepatosteatosys in mice. Some human studies showed that it stimulates oxidative metabolism (particularly hepatic fatty acid oxidation), can promote weight loss, and reduces blood cholesterol and inflammation. Moreover, *cis*-vaccenic (18:1 n-7) acid, also present in sea buckthorn oil, has been shown to reduce blood cholesterol, triacylglycerols and atherosclerosis in mice and was inversely associated with diabetes in humans [50]. It has already been established that sea buckthorn oil is a promising alternative in the treatment of gastro-intestinal disorders (liver diseases, ulcers and gastritis), of skin and mucosa ulcerative disorders, and it is also known for lowering blood cholesterol and for its anti-inflammatory properties [35]. The synergy between the active components of the oil, associated with their high bioaccessibility could explain the multitude of pharmacological effects of sea buckthorn fruits and oil.

## 4. Conclusions

The number of studies regarding xanthophyll bioaccessibility is limited, compared to carotenes. Therefore, in this study, we aimed at validating sea buckthorn oil as a valuable source of bioaccessible xanthophylls. Overall, using a plant-based oil-in-water emulsion as a delivery system of carotenoids to improve the bioaccessibility of these naturally occurring bioactive compounds has been proven a good strategy. Not only were the total and individual carotenoid bioaccessibilities higher than sea buckthorn oil, but also the hydrolysis efficiency after the introduction of CEL in the physiologically standardized INFOGEST protocol. Furthermore, the ability of CEL to cleave zeaxanthin esters (mainly ZDP) along with pancreatic lipase in the in vitro digestion model was highlighted. Under optimized conditions, we obtained the highest bioaccessibility of free zeaxanthin of 64.55% after the in vitro digestion of the oil-in-water emulsion, upon supplementation with CEL.

It should be mentioned that the complex processes occurring during human digestion are not completely simulated during in vitro digestion, with the latter being successfully used for preliminary studies having a specific endpoint (for example the bioaccessibility of bioactive compounds from different food matrices) that can be further investigated in more detail using in vivo methods.

## Figures and Tables

**Table 1:** Carotenoid composition of sea buckthorn oil.

Peak No.	Compound	UV-VIS Maxima	Carotenoid Concentration	
			mg/100 g ^a^	%
1	Lutein	421,445, 473	8.95 ± 0.22	2.95
2	Zeaxanthin	426, 450, 475	9.74 ± 0.90	3.21
3	15-*cis*-β-carotene	337, 420, 449, 472	6.13 ± 0.20	2.03
4	*all-trans-*β-carotene	421, 452, 478	10.61 ± 0.41	3.50
5	α-carotene	422, 444, 473	5.76 ± 0.49	1.90
6	Zeaxanthin myristate (C_14:0_)	426, 450, 475	8.71 ± 0.61	2.88
7	Zeaxanthin palmitate (C_16:0_)	426, 450, 475	10.69 ± 0.71	3.53
8	β-cryptoxanthin myristate (C_14:0_)	428, 451, 476	10.00 ± 0.89	3.30
9	Lutein oleate (C_18:1_)	421, 446, 474	11.88 ± 0.80	3.92
10	β-cryptoxanthin palmitate (C_16:0_)	428, 451, 476	18.27 ± 0.42	6.03
11	Zeaxanthin dimyristate (C_14:0_, C_14:0_)	426, 450, 475	21.26 ± 0.15	7.02
12	Zeaxanthin palmitate-palmitoleate (C_16:0_, C_16:1_)	427, 450, 476	19.76 ± 0.22	6.52
13	Zeaxanthin myristate-palmitate (C_14:0_, C_16:0_)	426, 450, 475	46.08 ± 0.92	15.22
14	Zeaxanthin dipalmitate (C_16:0_, C_16:0_)	427, 450, 476	114.98 ± 1.03	37.97
Carotenoid mono-esters			59.55 ± 3.39	19.67
Carotenoid di-esters			202.07 ± 2.32	66.73
Total identified carotenoid content			302.82 ± 7.84	

^a^ Average of triplicate samples.

**Table 2:** Fatty acid composition of sea buckthorn oil.

Fatty Acid	% of Total Fatty Acids *
Myristic acid (14:0)	0.31 ± 0.06
Palmitic acid (16:0)	49.42 ± 0.67
Palmitoleic acid (16:1 n-7)	28.04 ± 0.05
Stearic acid (18:0)	0.61 ± 0.10
Oleic acid (18:1 n-9)	11.74 ± 0.13
*Cis*-Vaccenic acid (18:1 n-7)	4.47 ± 0.12
Linoleic acid (18:2 n-6)	4.13 ± 0.13
α-linolenic acid (18:3 n-3)	1.27 ± 0.06
SFA	50.34 ± 0.83
MUFA	44.24 ± 0.30
PUFA	5.40 ± 0.18

* mean ± SD (*n* = 3). SFA: saturated fatty acids. MUFA: mono-unsaturated fatty acids. PUFA: poly-unsaturated fatty acids.
